# Effectiveness of Dietary Interventions in Prevention and Treatment of Iron-Deficiency Anemia in Pregnant Women: A Systematic Review of Randomized Controlled Trials

**DOI:** 10.3390/nu14153023

**Published:** 2022-07-23

**Authors:** Dominika Skolmowska, Dominika Głąbska, Aleksandra Kołota, Dominika Guzek

**Affiliations:** 1 Department of Dietetics, Institute of Human Nutrition Sciences, Warsaw University of Life Sciences (SGGW-WULS), 159C Nowoursynowska Street, 02-776 Warsaw, Poland; dominika_skolmowska@sggw.edu.pl (D.S.); aleksandra_kolota@sggw.edu.pl (A.K.); 2 Department of Food Market and Consumer Research, Institute of Human Nutrition Sciences, Warsaw University of Life Sciences (SGGW-WULS), 159C Nowoursynowska Street, 02-776 Warsaw, Poland; dominika_guzek@sggw.edu.pl

**Keywords:** anemia, pregnant, pregnancy, iron, iron deficiency, iron intake, vitamin C intake, diet, nutrition, randomized controlled trials

## Abstract

Pregnant women are among the population groups most vulnerable to the development of anemia, as the overall iron requirement during pregnancy is significantly higher than in non-pregnant women. The aim of the systematic review was to assess the effectiveness of dietary interventions in the prevention and treatment of iron-deficiency anemia in pregnant women based on randomized-controlled trials. The systematic review was based on the PRISMA guidelines and is registered in the PROSPERO database (CRD42021261235). The search was conducted within PubMed and Web of Science databases for the period until June 2021. The included randomized controlled trials presented effectiveness of dietary interventions in prevention and treatment of iron-deficiency anemia in pregnant women. From the total number of 7825 screened records, the final number of seven studies were included in the systematic review. The procedure of screening, inclusion, reporting, and assessment of the risk of bias while using the revised Cochrane risk of bias tool for randomized trials was conducted by two independent researchers. The studies included in the systematic review were conducted in populations of anemic pregnant women, or mixed populations of anemic and non-anemic pregnant women. The interventions described within the studies were associated with including fortified products, regular products, or dietary counselling. They were based on providing an increased amount of iron, providing an increased amount of multiple nutrients, or general counselling only, while effectiveness was compared with effectiveness of the placebo, supplementation, or control group. The study duration was diversified from a few weeks to half a year or longer. The major biochemical measure assessed within the included studies was hemoglobin. All applied dietary interventions, based on providing increased amount of iron, providing increased amount of multiple nutrients, or general counselling only, were effective. The majority of included studies were assessed as ones of a medium risk of bias. For some studies a high risk of bias was indicated, which resulted from a risk of bias arising from the randomization process, due to deviations from the intended interventions, and in selection of the reported result. Considering this fact, more randomized controlled trials should be planned and conducted in a rigorous manner to confirm the formulated observations of effectiveness of the studied interventions based on providing an increased amount of iron, providing an increased amount of multiple nutrients, or general counselling only.

## 1. Introduction

According to the World Health Organization (WHO), anemia is a major global health problem [1], which affects nearly two billion people worldwide [2]. Among the most vulnerable population groups for its development are women of childbearing age and pregnant women [3], while anemia is defined for them as a hemoglobin concentration below 12.0 g/dL and below 11.0 g/dL [4], respectively.

Anemia in pregnant women correlates with adverse perinatal outcomes, including intrauterine growth retardation, premature labor, low birth weight, or neonatal anemia [5], and is responsible for maternal consequences too, including the increased risk of pre-eclampsia and postpartum depression [6]. Taking these serious health consequences into account, the WHO has indicated that among the Global Nutrition Targets which should be achieved by 2025, is a 50% reduction in anemia frequency in women of reproductive age [7].

Depending on the etiology, the anemia is classified as hypoproliferative microcytic (caused e.g., by iron deficiency), hypoproliferative normocytic (caused e.g., by chronic diseases and inflammatory process), hypoproliferative macrocytic (caused e.g., by folate and vitamin B12 deficiency), and hemolytic (caused by e.g., hemoglobinopathies or enzymopathies) [8], while the most common reason for anemia is iron deficiency [8]. This problem appears during pregnancy, as the overall iron requirement in this period is significantly higher than in non-pregnant women, despite the temporary absence of menstruation [9]. Iron needs increase meaningfully during pregnancy in order to compensate the iron loss at delivery and to meet increased demands of the fetoplacental unit [9]. Therefore, proper nutrition during pregnancy, including adequate iron intake, plays a crucial role in determining the long-term nutritional status of mother and unborn baby [10]. Moreover, it is suggested that the dietary inadequacy attributable to dietary habits during pregnancy is higher compared to any other stage of life [11,12]. 

Oral iron supplementation is the first-line management of iron-deficiency anemia in pregnancy [13]. However, for the administration of oral iron supplements in pregnant women, its compliance is becoming a problem, due to either early pregnancy morning sickness, or late pregnancy constipation and abdominal discomfort, which may be intensified by applied iron supplementation and may discourage women from taking supplements [14]. Taking this into account, dietary interventions aiming at improving diet quality, as well as increasing food diversity, may be the most desirable and effective interventions [15]. Food fortification, in particular, is indicated by WHO as a cost-effective public health strategy to obtain relatively quick improvement in nutritional status of a population [16]. 

Considering the above-mentioned points, it should be indicated that objective assessment of effectiveness of dietary interventions in the prevention and treatment of anemia in pregnant women is needed. Therefore, the aim of the present systematic review was to assess the effectiveness of dietary interventions in the prevention and treatment of iron-deficiency anemia in pregnant women based on randomized-controlled trials.

## 2. Materials and Methods

### 2.1. Study Design

The process of literature screening, including, and reporting was based on the guidelines of the Preferred Reporting Items for Systematic Reviews and Meta-Analyses (PRISMA) [17]. The peer-reviewed randomized controlled trials were included if published by June 2021 and included in PubMed and Web of Science databases. The systematic review was registered in the International Prospective Register of Systematic Reviews (PROSPERO) database (CRD42021261235). 

### 2.2. Inclusion and Exclusion Criteria 

The randomized controlled trials were included in the systematic review if they assessed the effectiveness of any dietary intervention (compared with the other dietary approach or placebo or control or supplementation) in either the prevention or treatment of iron-deficiency anemia in pregnant women.

The inclusion criteria were as follows:

(1) Research study;
(2) Randomized controlled trial;
(3) Study conducted in a group of pregnant women;
(4) Study conducted in a group of subjects with either adequate iron status (prevention of iron-deficiency anemia) or inadequate iron status defined (treatment of iron-deficiency anemia);
(5) Dietary intervention applied within the study, using either regular food products, or fortified food products;
(6) The effectiveness of dietary intervention in either prevention or treatment, assessed within the study, using any biochemical measure of anemia/iron stores;
(7) The effectiveness of dietary intervention, assessed within the study, compared with the effectiveness of the other dietary approach or placebo or control or supplementation;
(8) Full text of the study published in English, in a peer-reviewed journal.

The exclusion criteria were as follows:

(1) Study conducted in animal model;
(2) Study conducted in a mixed population (e.g., pregnant and nonpregnant women), unless presenting results separately for sub-groups;
(3) Study conducted in a group of subjects with any condition which may influence iron status (e.g., celiac disease, bariatric surgery);
(4) Study conducted in a group of subjects with any eating disorder which may influence the reliability of results;
(5) Study conducted in a group of subjects with any intellectual disability which may influence the reliability of results;
(6) Applied dietary intervention not described within the study;
(7) The effectiveness of dietary intervention not defined (e.g., no baseline data presented), or influenced by any interfering factor applied within the study (e.g., pharmacological intervention, physical activity intervention).

No other criteria associated with concurrent diseases and conditions, except for those which may influence iron status or influence the reliability of results, were set.

The criteria for a population, intervention/exposure, comparator, outcome, and study design (PICOS) [18] are presented in Table 1.

### 2.3. Searching Strategy

The literature search focused on studies available in PubMed and Web of Science databases. The strategy of the electronic searching within PubMed and Web of Science databases is presented in detail in Table 2.

**Figure 1:**
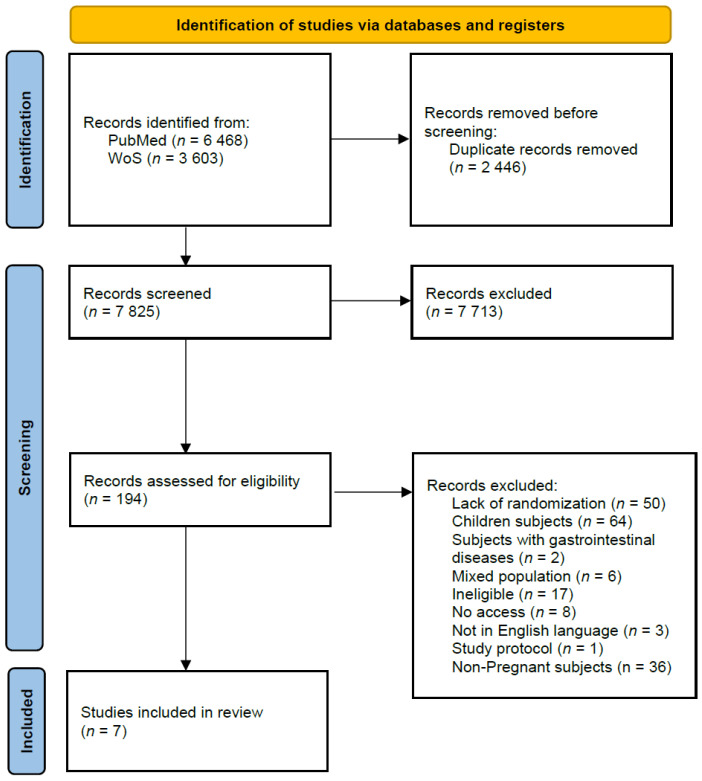
The identification, screening, and inclusion procedure of the studies available in PubMed and Web of Science databases.

The identification, screening, and inclusion procedure of the studies available in PubMed and Web of Science databases is presented in Figure 1. The whole process was conducted by two independent researchers, initially based on the title and abstract, followed by the procedure based on the full text of the study. In case of any disagreement between assessing researchers, the third researcher was consulted to assess the issue. The full texts of the studies were obtained from electronic databases, or the university library, but if they were not available, the corresponding author was contacted to provide full text.

### 2.4. Procedure of Data Extraction 

The extraction of data was conducted by two independent researchers. In case of any disagreement between assessing researchers, the third researcher was consulted to assess the issue. All necessary information was obtained from full texts of the studies, but if they were not available, the corresponding author was contacted to provide information (six emails sent, information presented as data provided on request).

The following information was extracted from the included studies:

(1) Basic characteristics (authors of the study, compared interventions, studied group of women, country/location, time);
(2) Characteristics of the participants (number of participants, age, inclusion criteria, exclusion criteria);
(3) Description of the intervention applied (characteristics of applied intervention, iron intake within diet, vitamin C intake within diet, study duration, biochemical measure);
(4) Observations and conclusions formulated within the randomized controlled trials included in the systematic review.

To assess the quality of the included randomized controlled trials, the risk of bias was assessed [19], while using the Cochrane risk of bias tool for randomized trials, and the RoB 2 tool (7.0) [20]. The included randomized controlled trials were assessed in the following domains: risk of bias arising from the randomization process, risk of bias due to deviations from the intended interventions, risk of bias due to missing outcome data, risk of bias in measurement of the outcome, and risk of bias in selection of the reported result, followed by the assessment of the overall risk of bias [21].

## 3. Results

The basic characteristics of the randomized controlled trials included in the systematic review are presented in Table 3. The randomized controlled trials included in the systematic review were conducted in populations of anemic women [22,23], or mixed populations of anemic and non-anemic women [24,25,26,27,28], from Asian countries: India [22,27], Vietnam [25], Indonesia [26], Cambodia [28], or African countries: Tanzania [24] and Egypt [23]. The interventions described within the studies included fortified products [24,25,28], regular products [26], or dietary counselling [22,23,27,28], while effectiveness was compared with the effectiveness of a placebo product [24,25], various supplementations [23,25], or a control group without any intervention [22,26,27,28].

The characteristics of the participants of the randomized controlled trials included in the systematic review is presented in Table 4. The randomized controlled trials included in the systematic review were conducted in medium-size populations of less than 100 participants [23,27], or large populations of more than 100 participants [22,24,25,26,28]. Within the inclusion and exclusion criteria there were indicated characteristics such as specific hemoglobin blood level [22,23,24,25], specific age [23,25,26,27,28], and specific weeks of gestation [22,23,24,26,27,28].

The description of the intervention applied within the randomized controlled trials included in the systematic review is presented in Table 5. The applied dietary interventions were based on providing increased amount of iron [23,25], providing increased amount of multiple nutrients [22,24,26,28], or general counselling only [27]. The study duration was diversified from few weeks [23,24], through few months [22,25,27], to half a year or longer [28]. The major biochemical measure assessed within the included studies was hemoglobin [22,23,24,25,26,27,28], but in some studies ferritin [23,24,26], transferrin saturation and receptor [25,26], serum and body iron concentrations [23,26], mean corpuscular volume (MCV) [23], mean corpuscular hemoglobin (MCH) [23], or total iron binding capacity were also assessed [23].

The observations and conclusions formulated within the randomized controlled trials included in the systematic review are presented in Table 6. Based on the results presented by authors of the randomized controlled trials included in the systematic review, as well as on the conclusions formulated by them, it may be indicated that all the included studies presented effective dietary interventions, independently for the studied population of either anemic women [22,23], or a combined population of anemic and non-anemic women [24,25,26,27,28]. 

The summary of conclusions formulated within the randomized controlled trials included in the systematic review is presented in Table 7. Based on the observations and conclusions formulated within the included studies, it was stated that all applied dietary interventions, based on providing an increased amount of iron [23,25], an increased amount of multiple nutrients [22,24,26,28], or general counselling only [27] were effective, and the formulated conclusions supported all approaches listed. 

The assessment of the risk of bias within the randomized controlled trials included in the systematic review, conducted using the revised Cochrane risk of bias tool for randomized trials, is presented in Table 8. The majority of included studies were assessed as ones of a medium risk of bias [22,23,24,27], which resulted from some concerns associated with risk of bias arising from the randomization process [22,24,27] and risk of bias in selection of the reported result [22,23,27]. At the same time, for some studies a high risk of bias was indicated [25,26,28], which resulted from a high risk of bias arising from the randomization process [26], a high risk of bias due to deviations from the intended interventions [28], and a high risk of bias in the selection of the reported result [25]. 

## 4. Discussion

In the present systematic review, it was revealed that all assessed dietary interventions turned out to be effective both in prevention and treatment of anemia in the group of pregnant women. In terms of risk of bias, the majority of included studies were characterized as studies of a medium risk of bias [22,23,24,27], but a high risk of bias was indicated for some studies [25,26,28]. It resulted from the randomization process, due to deviations from the intended interventions, and in the selection of the reported result. 

There are a number of studies which assess the effectiveness of dietary interventions in terms of prevention and treatment of anemia in the group of non-pregnant women [29,30,31,32,33,34,35]. Similarly, as in the case of studies conducted in the group of pregnant women, studies on non-pregnant women also include increased supply of iron [29,30,31] and improving iron bioavailability, by controlling the supply of other nutrients [32,33] and combining an increased iron supply along with increasing its absorption [34,35]. However, the obtained results are not as unambiguous as in the studies included in this systematic review. Some dietary interventions were proven to be insufficient to improve iron status among non-pregnant women [30,32], while in this review all studied interventions were effective in the prevention and dietary treatment of anemia in pregnant women. 

Pregnancy is a time of rapid physiological changes during which nutritional requirements increase to maintain maternal metabolism, as well as support fetal growth and development [36]. Adequate dietary habits play a crucial role in determining the nutritional status of the mother and fetus [37]. As one of the major health problems among pregnant women is anemia [38], attention should be especially paid to a properly balanced diet. Pregnant women are advised to increase their overall food intake by consumption [39], while additional amount of energy may help them meet an increased requirement for iron, which is estimated as 27 mg/day in pregnancy [40]. This need for additional iron and energy supply during pregnancy may explain the observed effectiveness of studied interventions, as nonheme iron absorption during pregnancy increases as gestation progresses [41], which is associated with a maternal red blood cell mass increase, as well as placental and fetal growth accelerating [42]. Considering this fact, the interventions applied during pregnancy may be more effective than those applied in non-pregnant women.

Currently, daily iron supplementation is recommended by the WHO as a part of antenatal care to reduce the risk of maternal anemia and risk of low birth weight [43]. However, it is indicated that the proper compliance to iron supplementation may be challenging for pregnant women, as in the study of Fouelifack et al. [44], where 56% of women were found to be low-compliant with iron supplementation, while the main reasons for non-adherence were side effects, forgetting, and the inaccessibility of iron supplements. Therefore, it seems that dietary interventions may be a promising opportunity for pregnant women to improve their iron status, which in the presented systematic review were revealed to be effective.

Three main dietary strategies are pointed out as those which can manage anemia and improve iron status in pregnant women. The first approach is dietary improvement which aims at increasing iron intake by the selection of iron-containing food products, such as meat and fish, legumes, and green-leafy vegetables [45]. As cereal products may provide a high amount of iron in the diet of young women, their intake should be increased as well [46]. However, efforts should also be focused on iron bioavailability to maximize its absorption by providing substantial amounts of iron absorption enhancers within a meal, such as vitamin C and meat, and reducing the intake of iron inhibitors, such as phytates, polyphenols, and calcium [46,47]. Such approach is commonly applied in the studies, as most of the assessed studies in this systematic review were concerned with increasing the amount of multiple nutrients, including iron, but also other nutrients [22,24,26,28]. Another strategy to address anemia is iron fortification of staple foods, including wheat, maize, and rice [48]. Iron fortification according to the dietary habits of the affected population group is regarded as the most cost-effective long-term approach to reduce the prevalence of anemia [49], while food vehicles which are used should be affordable and consumed regularly among the targeted population [50]. Such strategy seems to be especially promising for low income countries with a serious problem of inadequate intake and food supply [51]. The last possible strategy of anemia management is nutritional counselling. In the presented systematic review, a study in which only counselling was used turned out to be effective in anemia prevention and management [27]. According to the WHO, pregnant women are recommended to have counselling about healthy eating and keeping physically active during pregnancy [52]. Taking this into account, tailoring nutrition education and counselling to the cultural and local context about diet in pregnancy, including adequate iron intake in anemia prevention and treatment, should be an area of particular interest [53].

In spite of the fact that the conducted systematic review provided some important conclusions associated with the effectiveness of dietary interventions in the prevention and treatment of iron-deficiency anemia in pregnant women based on the randomized-controlled trials, some limitations must be indicated. The major problem is associated with a limited number of studies conducted so far. At the same time, some sources of bias were revealed within the conducted studies, which reduces the possibility to draw conclusions. Last but not least, the conclusions of some studies addressed improvements in the assessed parameters, but not the clinical relevance of the observed changes, which may have been within the range of laboratory error, so interpretation of some results may be challenging.

## 5. Conclusions

All dietary interventions studied within randomized controlled trials included in the systematic review, based on providing an increased amount of iron, providing an increased amount of multiple nutrients, or on general counselling only, were effective in the prevention or treatment of anemia in pregnant women. The majority of included studies were assessed as ones of a medium risk of bias, but a high risk of bias for some studies was indicated, which resulted from a risk of bias arising from the randomization process, due to deviations from the intended interventions, and in selection of the reported result. Considering this fact, more randomized controlled trials should be planned and conducted in a rigorous manner to confirm the formulated observations of effectiveness of the studied interventions based on providing an increased amount of iron, providing an increased amount of multiple nutrients, or general counselling only.

## Figures and Tables

**Table 1:** The criteria for a population, intervention/exposure, comparator, outcome, and study design (PICOS).

PICOS	Inclusion Criteria	Exclusion Criteria
Population	Anemic and non-anemic pregnant women	Pregnant women with diseases and conditions, which may influence either iron status or reliability of results
Intervention/exposure	Dietary intervention applied to prevent or treat anemia	Undefined dietary intervention
Comparison	Effectiveness of dietary intervention vs. effectiveness of supplementation/placebo/control, or the other dietary approach	Effectiveness of dietary intervention not defined, or influenced by interfering factors
Outcome	Biochemical parameters of anemia/iron stores/iron status	Biochemical parameters of anemia/iron stores/iron status for a mixed population of pregnant and non-pregnant women
Study design	Randomized controlled trials	Results not published peer-reviewed journals, not published in English, and retracted articles

**Table 2:** The strategy of the electronic searching within PubMed and Web of Science databases.

Database	The Detailed Strategy of the Electronic Searching
PubMed	(“anaemia” [Title/Abstract] OR “anemia” [Title/Abstract] OR “anaemic” [Title/Abstract] OR “anemic” [Title/Abstract] OR “low haemoglobin” [Title/Abstract] OR “iron status” [Title/Abstract]) AND (“iron” [Title/Abstract]) AND (“nutrition” [Title/Abstract] OR “diet” [Title/Abstract] OR “diets” [Title/Abstract] OR “nutritional” [Title/Abstract] OR “dietary” [Title/Abstract])
Web of Science	AB = (anaemia OR anemia OR anaemic OR anemic OR low haemoglobin OR iron status) AND AB = (iron) AND AB = (nutrition OR diet OR diets OR nutritional OR dietary)

**Table 3:** The basic characteristics of the randomized controlled trials included in the systematic review [22,23,24,25,26,27,28].

Ref.	Authors, Year	Compared Interventions	Studied Group of Women	Country/Location	Time
[24]	Makola et al., 2003	Diet with fortified beverage vs. diet with placebo beverage	Pregnant anemic and non-anemic women	Tanzania/Mpwapwa and Kongwa districts	August–October 1999
[25]	Hoa et al., 2005	Diet with milk fortified with iron vs. diet with nonfortified milk vs. supplement vs. placebo	Pregnant anemic and non-anemic women	Vietnam/Thai Binh province	1996–1997
[22]	Susheela et al., 2010	Diet vs. control	Pregnant anemic women	India/Delhi	Beginning: 2005; for 2.5 years
[26]	Wijaya-Erhardt et al., 2011	Diet vs. control	Pregnant anemic and non-anemic women	Indonesia/Central Java province	November 2007–October 2008
[27]	Shivalli et al., 2015	Diet vs. control	Pregnant anemic and non-anemic women	India/Varanasi district	May 2010–April 2011
[28]	Janmohamed et al., 2016	Diet with corn soya blend vs. control	Pregnant anemic and non-anemic women	Cambodia/Kampong Chhnang province	Recruitment: August 2011–June 2012
[23]	Darwish et al., 2018	Diet with lactoferrin vs. total dose infusion of low-molecular weight iron dextran	Pregnant anemic women	Egypt/Assiut	September 2015–October 2017

**Table 4:** The characteristics of the participants of the randomized controlled trials included in the systematic review [22,23,24,25,26,27,28].

Ref.	Number of Participants	Age (Mean/Median/Range)	Inclusion Criteria	Exclusion Criteria
[24]	259	25.4 years	Pregnancy; 12–34 week of gestation; attending prenatal clinics in the hospitals and surrounding health centers of Mpwapwa and Kongwa districts	Hemoglobin concentration of <80 g/L; serious medical condition; complication of pregnancy such as cardiac disease, pneumonia, and threatened abortion
[25]	168	25.0–25.8 years, depending on group	Pregnancy; age of 20–32 years; no more than two prior pregnancies; hemoglobin > 70 g/L	Stillbirths, premature births, or hemorrhage in previous pregnancies; manifestations of chronic or infectious diseases, including hookworm infection; planned travel or plans to move out of the area during the study period
[22]	205	Not specified	Pregnancy; anemia (hemoglobin 50–90 g/L); urinary fluoride > 1.0 mg/L	Gestation > 20 weeks; diabetes; tuberculosis; bleeding during pregnancy; high blood pressure; HIV/AIDS; malaria; other medical problems
[26]	227	15–49 years	Pregnancy; age of 15–49 years; 12–20 weeks of gestation; predicted singleton neonates	Severe maternal illness
[27]	86	22.9–23.2 years, depending on group	Pregnancy; age of 15–45 years; a history of amenorrhea; 13–28 week of gestation	Acute illness; severe medical or obstetrical complications; multiple pregnancy; gestational diabetes; not staying for a minimum period of 12 weeks in the study area
[28]	495	26.2–26.9 years, depending on group	Pregnancy; age of ≥18 years; being in the first trimester of pregnancy; planning to stay in the home village for the duration of the pregnancy	Fetal loss; migration
[23]	93	27.3–29.5 years, depending on group	Pregnancy; age of ≥18 years; 14–28 weeks of gestation; iron-deficiency anemia (hemoglobin level of 70–100 g/L)	Anemia predominantly caused by factors other than iron-deficiency (e.g., anemia with untreated B12 or folate deficiency, hemolytic anemia); iron overload or disturbances in utilization of iron (e.g., hemochromatosis and hemosiderosis); decompensated liver cirrhosis and active hepatitis; active acute or chronic infections; rheumatoid arthritis with symptoms or signs of active inflammation; history of multiple allergies; gastrointestinal tract diseases; known hypersensitivity to parenteral iron or any recipients in the investigational drug products; receiving erythropoietin treatment within 8 weeks prior to the screening visit or other iron treatment or blood transfusion within 4 weeks prior to the screening visit

**Table 5:** The description of the intervention applied within the randomized controlled trials included in the systematic review [22,23,24,25,26,27,28].

Ref.	Characteristics of Applied Intervention	Iron Intake within Diet	Vitamin C Intake within Diet	Study Duration	Biochemical Measure
[24]	(1) Diet with fortified beverage: orange-flavored micronutrient-fortified powdered beverage mix containing 11 micronutrients (iron, iodine, zinc, vitamin A, vitamin C, vitamin E, riboflavin, niacin, vitamin B6, folic acid, and vitamin B12) (176 kcal/day; iron: 10.8 mg/day; vitamin C: 144 mg/day); (2) Diet with placebo beverage (176 kcal/day)	Not specified	Not specified	8 weeks	Hemoglobin, serum ferritin
[25]	(1) Diet with milk powder fortified with iron: 400 mL of milk fortified with iron (15 mg/day), fortified with vitamin C and folic acid; (2) Diet with iron nonfortified milk powder: 400 mL of nonfortified milk, fortified with vitamin C and folic acid; (3) Supplement: daily iron-folic acid supplement in pill form (60 mg/day); 250 μg of folic acid; (4) Placebo: placebo tablet	At baseline: 9.7–10.3 mg/day, depending on group (no differences)	At baseline: 41.1–50.4 mg/day, depending on group (no differences)	16 weeks	Hemoglobin, transferrin saturation
[22]	(1) Diet: counselling based on intake of calcium, iron, folic acid, vitamins C, E and other antioxidants through dairy products, vegetables, and fruits, accompanied by removal of fluoride from ingestion through drinking water, food, and other sources; (2) Control: no dietary intervention	Not specified	Not specified	20 weeks	Hemoglobin
[26]	(1) Diet: 600 g of tempeh, 30 g of meat, 30 g of dry anchovies, 30 g of chicken liver, 350 g of guava, 300 g of papaya, 100 g of orange provided weekly as a supplementary products (providing 3.97 mg of iron and 173 mg of vitamin C per day); free access to receive tablets containing 60 mg of Fe and 250 mg of folic acid; (2) Control: no dietary intervention; free access to receive tablets containing 60 mg of Fe and 250 mg of folic acid	Not specified	Not specified	Not specified	Hemoglobin, ferritin, transferrin receptor, body iron concentration
[27]	(1) Diet: Trials of Improved Practices (TIPs) applied through 3 home visits (assessment, negotiation, and evaluation) to interview, counsel and assess the results of implementing novel dietary practices; (2) Control: no dietary intervention applied within TIPs (2 home visits for assessment and evaluation)	At baseline: Diet: 19 ± 7.02 mg/day; control: 19.05 ± 6.63 mg/day After intervention: Diet: 21.58 ± 7.25 mg/day; control: 19.96 ± 6.59 mg/day	Not controlled *	12 weeks	Hemoglobin
[28]	(1) Diet: counselling focused on best practices related to diet, anemia prevention and management; Corn Soya Blend Plus supplements (CSB Plus) provided from the first trimester to delivery (6.75 kg of CSB Plus and 300 mL of vitamin A- and vitamin D-fortified palmolein oil to be added during cooking, monthly—daily ration of 200 g of CSB Plus and 10 mL of oil—850 kcal, 13 mg of iron, 200 mg of vitamin C); receiving daily tablets containing iron (60 mg) and folic acid (400 mg) and if anemic—2 iron-folic acid tablets per day for 14 days; (2) Control: counselling focused on best practices related to diet, anemia prevention and management; no CSB Plus provided; receiving daily tablets containing iron (60 mg) and folic acid (400 mg) and if anemic—2 iron-folic acid tablets per day for 14 days	Not specified	Not specified	6–8 months	Hemoglobin
[23]	(1) Diet with lactoferrin: pineapple flavored lactoferrin oral sachets (100 mg) two times per day for 4 weeks accompanied with health education (including treatment for anemia during pregnancy); avoiding coffee and tea particularly immediately after meals; increasing dietary intake of iron-rich food and vitamin C-rich food; (2) Total dose infusion of low-molecular weight iron dextran (individually calculated using Ganzoni formula); avoiding coffee and tea, particularly immediately after meals; increasing dietary intake of iron-rich food and vitamin C-rich food	Not controlled *	Not controlled *	4 weeks	Hemoglobin, mean corpuscular volume (MCV), mean corpuscular hemoglobin (MCH), serum iron, ferritin, and total iron binding capacity

* Data provided on request.

**Table 6:** The observations and conclusions formulated within the randomized controlled trials included in the systematic review [22,23,24,25,26,27,28].

Ref.	Observations	Conclusions
[24]	The supplement resulted in a 4.16 g/L increase in hemoglobin concentration and a 3 µg/L increase in ferritin and reduced the risk of anemia and iron deficiency anemia by 51 and 56%, respectively. The risk of iron deficiency was reduced by 70% among those who had iron deficiency at baseline and by 92% among those who had adequate stores.	The micronutrient-fortified beverage may be a useful and convenient preventative measure, one that could help improve the nutritional status of women both before and during pregnancy and thereby help avoid some of the potential maternal and fetal consequences of micronutrient deficiencies.
[25]	After the 16th week of intervention, the changes in hemoglobin concentrations in both treatment groups (the iron-fortified milk and the iron tablet groups) were not significantly different (−0.5 ± 0.9 and −0.3 ± 0.9 g/L, respectively), but the changes were significantly greater in the nonfortified milk and placebo groups (−1.2 ± 0.9 and −1.1 ± 0.8 g/L, respectively; *p* < 0.01). The change in transferrin saturation in the iron-fortified milk group (3.4 ± 12.9%) was greater than that in the placebo and nonfortified milk groups (−10.1 ± 9.8% and −11.6 ± 10.7%, respectively) (*p* < 0.01).	Applying iron-fortified milk and the iron tablets may prevent deterioration of iron status.
[22]	An increase in hemoglobin upon nutritional intervention in 73% during the 1st trimester and in 83% during the 2nd trimester of pregnancy has been recorded.	An intervention approach has scope for reducing anemia in pregnancy.
[26]	At near term, mean hemoglobin, ferritin and body iron decreased, whereas mean transferrin receptor increased significantly in both groups. The mean changes in iron status were similar in both groups. In Fe-deficient women, consumption of an optimized diet was associated with smaller decreases in hemoglobin (1.02 (95% CI 0.98, 1.07) g/L; *p* = 0.058), ferritin (1.42 (95% CI 1.16, 1.75) µg/L; *p* = 0.046) and body iron (2.57 (95% CI 1.71, 3.43) mg/kg; *p* = 0.073) concentrations, compared with a state of no intervention. Fe-deficient women at baseline benefited more from supplementary food compared with Fe-replete women.	Daily supplementary food containing tempeh and vitamin C-rich fruits during pregnancy might have positive effects on maternal iron deficiency.
[27]	At the end, mean hemoglobin levels were 115 ± 12.4 g/L and 103.7 ± 13.8 g/L in the TIPs group and control group, respectively. The prevalence of anemia was reduced by half in the TIPs group and increased by 2.4% in the control group.	Trials of Improved Practices (TIPs) were found to be an effective approach to improve the nutritional status of pregnant women in the study area.
[28]	Significant reductions were observed in anemia at 36–38 week (OR = 0.51; 95% CI: 0.34, 0.77).	In Cambodian women, Corn Soya Blend Plus consumed during pregnancy significantly reduced maternal anemia in late gestation in comparison with women consuming a normal diet.
[23]	There was no statistically significant difference in mean hemoglobin level improvement in both groups after one month of therapy. However, MCV and MCH improved significantly more in group receiving infusions of iron dextran than diet with lactoferrin while iron indices (serum iron and serum ferritin) were significantly more in group receiving diet with lactoferrin than group receiving infusions of iron dextran.	Pineapple flavored lactoferrin oral sachets plus health education can be widely used as an alternative to total dose infusion iron dextran supplementation due to clinical as well as laboratory improvement of iron-deficiency anemia during pregnancy after one month of treatment.

**Table 7:** The summary of conclusions formulated within the randomized controlled trials included in the systematic review [22,23,24,25,26,27,28].

Dietary Approach	Ref.	Group of Studied Women	Conclusion *
Providing an increased amount of iron	[25]	Anemic and non-anemic women	Supporting
	[23]	Anemic women	Supporting
Providing an increased amount of multiple nutrients	[24]	Anemic and non-anemic women	Supporting
	[22]	Anemic women	Supporting
	[26]	Anemic and non-anemic women	Supporting
	[28]	Anemic and non-anemic women	Supporting
General counselling only	[27]	Anemic and non-anemic women	Supporting

* The assessment based on conclusions formulated within the study by the authors—defined as supporting or not supporting applied dietary intervention, based on the assessed biochemical measures.

**Table 8:** The assessment of the risk of bias within the randomized controlled trials included in the systematic review conducted using the revised Cochrane risk of bias tool for randomized trials [22,23,24,25,26,27,28].

Ref.	Ref.	D1	D2	D3	D4	D5	Overall Bias
Providing an increased amount of iron	[25]	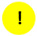	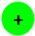	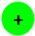	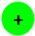	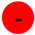	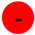
	[23]	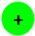	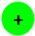	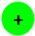	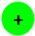	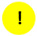	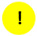
Providing an increased amount of multiple nutrients	[24]	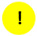	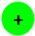	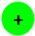	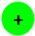	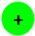	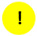
	[22]	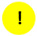	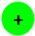	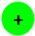	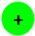	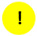	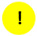
	[26]	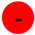	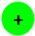	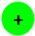	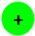	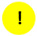	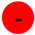
	[28]	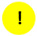	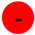	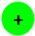	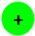	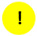	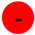
General counselling only	[27]	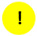	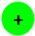	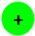	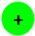	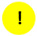	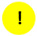

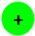
—Low risk; 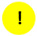
—Some concerns 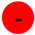
—High risk; Domains as follows: D1—risk of bias arising from the randomization process; D2—risk of bias due to deviations from the intended interventions; D3—risk of bias due to missing outcome data; D4—risk of bias in measurement of the outcome; D5—risk of bias in selection of the reported result.
